# Clinical Significance of Serum Soluble T Cell Regulatory Molecules in Clear Cell Renal Cell Carcinoma

**DOI:** 10.1155/2014/396064

**Published:** 2014-06-25

**Authors:** Akinori Masuda, Kyoko Arai, Daisaku Nishihara, Tomoya Mizuno, Hideo Yuki, Tsunehito Kambara, Hironori Betsunoh, Hideyuki Abe, Masahiro Yashi, Yoshitatsu Fukabori, Ken-Ichiro Yoshida, Takao Kamai

**Affiliations:** Department of Urology, Dokkyo Medical University, 880 Kitakobayashi, Mibu-machi, Shimotsuga-gun, Tochigi 321-0293, Japan

## Abstract

To clarify the role of serum soluble T cell regulatory molecules in clear cell renal cell carcinoma (CCRCC), we measured the serum levels of soluble interleukin-2 receptor (sIL-2R), soluble B7-H3 (sB7-H3), and soluble cytotoxic T lymphocyte associated antigen-4 (sCTLA-4) in 70 CCRCC patients and 35 healthy controls. We investigated correlations between the serum levels of these soluble T cell regulatory molecules and the pathological grade, clinical stage, and prognosis of CCRCC. We also assessed the relations among each of these soluble molecules. As a result, the serum level of sIL-2R was significantly higher in CCRCC patients than in healthy controls (*P* < 0.05). In addition, elevation of serum sIL-2R was significantly correlated with the clinical stage (*P* < 0.001), and the survival of patients with high sIL-2R levels was shorter than that of patients with low sIL-2R levels (*P* < 0.05). Furthermore, the serum level of sB7-H3 was also significantly correlated with the clinical stage (*P* < 0.05), while the sIL-2R and sB7-H3 levels showed a positive correlation with each other (*R* = 0.550, *P* < 0.0001). These results indicate that the serum level of sIL-2R reflects tumor progression in CCRCC patients. In addition, the possibility was suggested that the IL-2/IL-2R and B7-H3 pathways may be involved in the progression of CCRCC.

## 1. Introduction

Cytokines that are secreted by tumor cells or by host cells play an important role as mediators and/or regulators of tumor-host interactions involved in the progression of malignancy. T lymphocytes are important to the development of host immune response to human tumors and their participation is most evident in tumors for immunotherapy. Interleukin-2 (IL-2) is thought of as a major cytokine involved in effector T (Teff) and regulatory T (Treg) cells proliferation and differentiation. Renal cell carcinoma (RCC) has long been thought of as an immunogenic tumor. Cesana et al. reported that the number of Treg cells was increased in patients with metastatic melanoma and RCC and that high-dose IL-2 therapy caused a significant decrease of Treg cells in patients who achieved an objective clinical response with this treatment [1]. On the other hand, recent studies have suggested that low-dose IL-2 therapy suppresses the immune system in patients with chronic graft-versus-host disease (GVHD) and hepatitis C virus (HCV) related vasculitis [2, 3], and these studies have also shown that low-dose IL-2 increases the number of Treg cells. Murphy suggested that there is dynamic interaction between activation and suppression of the immune system, with various cytokines being capable of exerting effects in either direction and even doing so simultaneously to some extent [4]. Bluestone reported that high-dose IL-2 causes a relative increase of the Teff population, whereas low-dose IL-2 preferentially induces the expansion of Treg cells [5]. In addition, Malek et al. reported that many of the key effects of IL-2 on Treg cells, but not Teff cells, require minimal IL-2 receptor (IL-2R) signaling, providing a therapeutic window to promote immune tolerance by using low-dose IL-2 to act specifically on Treg cells [4, 6].

IL-2 binds to a specific receptor, the IL-2R, on target cells to induce various biologic responses. The IL-2R consists of three subunits, which are IL-2R*α* (CD25), IL-2R*β* (CD122), and IL-2R*γ* (CD132) [7, 8]. IL-2R*β* and IL-2R*γ* are constitutively expressed on the surface of T cells, whereas IL-2R*α* expression only becomes prominent after T cell activation. It has been shown that the high affinity receptor for IL-2 consists of all three subunits combined noncovalently and that the *α*-chain is essential for formation of the high affinity receptor [9]. Once activated by IL-2, T cells start to synthesize IL-2R*α*, so its expression is an indicator of T cell activation. A truncated form of IL-2R*α*, which is known as soluble IL-2R (sIL-2R), only has an extramembrane domain and is also detected in the serum [10, 11]. Release of sIL-2R from T cells is associated with activation of T cells and appears to play an important role in regulating the immune response.

It is well known that full activation of T lymphocytes requires two signals [12]. The first signal is antigen recognition, which is mediated by binding of a major histocompatibility complex peptide to specific T cell receptors. Complete activation and differentiation of T cells also require the second signal, the so-called costimulatory stimulus. The best characterized of the costimulatory stimuli involves the CD28/B7 family of molecules. B7-H3 is a member of the B7 family, which comprises B7-H1, B7-H2, and B7-H4 and shows structural similarities to the classical costimulatory molecules B7-1 and B7-2 [13]. It was recently reported that B7-H3 strongly inhibits T cell activity via an IL-2-dependent mechanism [14]. Cytotoxic T lymphocyte associated antigen-4 (CTLA-4) is a member of the immunoglobulin (Ig) gene superfamily and is a B7-binding protein along with its homologue (CD28) [15]. CTLA-4 plays an important role in the downregulation of T cell responses, in T cell homeostasis, and in maintenance of peripheral tolerance. It is thought that CTLA-4 signaling blocks IL-2 production and IL-2R expression [16]. However, the relations among serum levels of sIL-2R, sB7-H3, and sCTLA-4 in patients with clear cell renal cell carcinoma (CCRCC) remain unknown. In the present study, we therefore measured the serum levels of sIL-2R, sB7-H3, and sCTLA-4 in CCRCC patients and evaluated correlations between these soluble T cell regulatory molecules and the tumor pathological grade, the clinical stage, and the prognosis. We also investigated the relations among these soluble immune molecules.

## 2. Patients and Methods

We studied 70 patients (52 men and 18 women) in whom CCRCC was diagnosed at our institution from 2002 to 2012. All of them underwent preoperative imaging (CT and/or MRI) for tumor staging. The postoperative follow-up period ranged from 3 to 119 months (median: 27 months). Surgery was performed before patients received any other therapy. Tumors were staged clinically according to the TNM classification [17]. The clinical characteristics of the 70 patients are summarized in Table 1. Thirty-five healthy volunteers (23 men and 12 women aged 22–75 years) were enrolled as the control group. Serum samples obtained from the 70 patients with CCRCC and 35 healthy controls were stored at −80°C until analysis. All samples were collected from the CCRCC patients before surgery. The serum level of sIL-2R was measured with a Quantikine ELISA Human IL-2 sR*α* Immunoassay kit (R&D systems, USA) using two monoclonal antibodies that recognized different epitopes of Tac antigen. Serum sB7-H3 was determined by using a Quantikine ELISA Human B7-H3 Immunoassay kit (R&D systems, USA), and serum sCTLA-4 was measured with a CTLA4 Human ELISA kit (Abnova, USA). This study was conducted in accordance with the Helsinki Declaration and Institutional Review Board approval was obtained, and each patient gave written informed consent. Statistical analysis was performed by using the Mann-Whitney *U* test to compare two groups and the Kruskal-Wallis test for comparisons among three or more groups. Cause-specific survival curves were drawn for patients with CCRCC by the Kaplan-Meier method and differences of survival were examined by the log-rank test. Spearman's rank correlation coefficient analysis was used to determine the relations of each immunological molecule. In all analyses, a *P* value of less than 0.05 was considered significant. All statistical analyses were performed with EZR (Saitama Medical center, Jichi Medical University, Japan) [18].

## 3. Results

### 3.1. Correlations between Soluble T Cell Regulatory Molecules and Clinicopathological Features

When the healthy controls and CCRCC patients were compared, the serum level of sIL-2R was significantly higher in the patients than in the healthy controls (*P* = 0.043). In contrast, the serum levels of sB7-H3 and sCTLA-4 were significantly higher in the controls than in the CCRCC patients (*P* < 0.0001 and *P* < 0.001), respectively (Table 2(a)). When the serum levels of sIL-2R, sB7-H3, and sCTLAS-4 were determined for CCRCC patients with tumors of each grade, differences among the tumor grades were not significant (Table 2(b)). When the serum sIL-2R level was determined for CCRCC patients with tumors of each clinical stage, there were significant differences of sIL-2R among the stages (*P* < 0.001). The difference of sIL-2R between Stage I and Stage IV was significant (*P* < 0.001), while the differences between Stage I and Stage II or Stage I and Stage III were not significant (*P* = 0.456 and *P* = 0.552), respectively. There were also significant differences of the serum sB7-H3 level among the clinical stages (*P* = 0.044). The difference of sB7-H3 between Stage I and Stage IV was significant (*P* = 0.049), while the differences between Stage I and Stage II or Stage I and Stage III were not significant (*P* = 0.505 and *P* = 0.585), respectively. In contrast, the serum level of sCTLA did not show any differences among the tumor stages (*P* = 0.179) (Table 2(c)).

### 3.2. Correlation with Cause-Specific Survival

**Figure 1:**
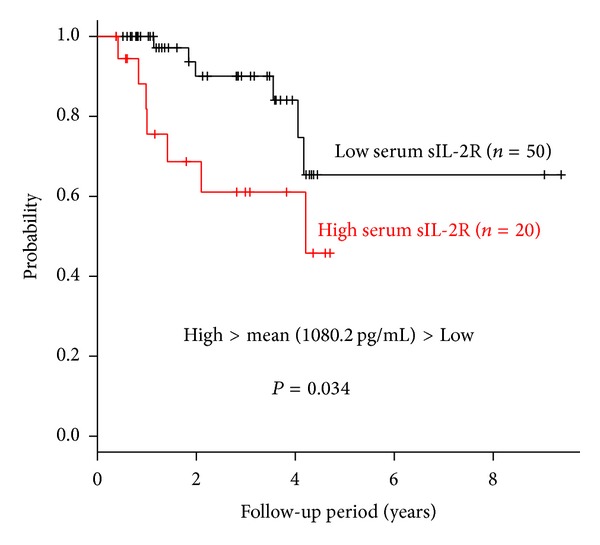
Cause-specific overall survival of 70 patients with clear cell renal cell carcinoma (CCRCC) stratified by the mean serum level of soluble interleukin-2 receptor (sIL-2R). Patients were divided into two groups (high and low) based upon whether the serum level of sIL-2R was above or below the mean value in all 70 patients. Cause-specific survival curves were drawn by the Kaplan-Meier method and differences of survival were examined by the log-rank test.

The CCRCC patients were divided into two groups (high or low) based upon whether each soluble T cell regulatory molecule was above or below the mean serum level for all 70 patients. When patients with low (*n* = 50) and high (*n* = 20) serum levels of sIL-2R were compared, their survival rate showed a significant difference by the log-rank test (*P* = 0.034) (Figure 1). In contrast, the survival of patients with low (*n* = 50) or high (*n* = 20) serum sB7-H3 levels did not differ significantly (*P* = 0.461). Survival of patients with low (*n* = 43) or high (*n* = 27) serum sCTLA-4 levels was also not significantly different (*P* = 0.964).

### 3.3. Correlations among Soluble T Cell Regulatory Molecules

**Figure 2:**
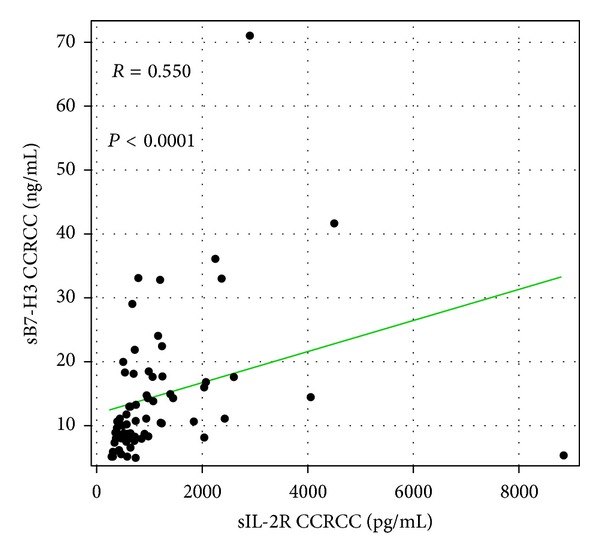
Spearman's rank correlation coefficient analysis of the relation among soluble interleukin-2 receptor (sIL-2R) and soluble B7-H3 (sB7-H3) in patients with clear cell renal cell carcinoma (CCRCC).

We analyzed the correlations among the serum levels of sIL-2R, sB7-H3, and sCTLA4 in the CCRCC patients and healthy controls. The serum level of sIL-2R was positively correlated with that of sB7-H3 in the CCRCC patients (*R* = 0.550, *P* < 0.0001) (Figure 2). However, the serum levels of sIL-2R and sCTLA-4 were not correlated in CCRCC patients (*R* = 0.157, *P* = 0.193). Serum sB7-H3 was also not correlated with sCTLA-4 in cancer patients (*R* = 0.049, *P* = 0.687). The serum level of sIL-2R was not correlated with that of sB7-H3 in healthy controls (*R* = 0.296, *P* = 0.084), and sIL-2R was also not correlated with sCTLA-4 (*R* = 0.300, *P* = 0.080). However, serum sB7-H3 was positively correlated with serum sCTLA-4 in the healthy controls (*R* = 0.685, *P* < 0.0001).

## 4. Discussion

In the present study, we observed that the serum level of sIL-2R was significantly higher in CCRCC patients than in the healthy controls. This increase of serum sIL-2R was significantly related to the clinical stage of the cancer and the survival of patients with high levels of serum sIL-2R was shorter than that of patients with low levels. Our results indicate that the pretreatment serum sIL-2R level of patients with CCRCC reflects tumor progression and that high levels of sIL-2R may have a negative prognostic impact. Previous studies have shown that elevation of serum sIL-2R has a negative prognostic effect in patients with various malignant tumors, such as malignant melanoma [19, 20], head and neck cancer [21], nasopharyngeal carcinoma [22], non-Hodgkin's lymphoma [23, 24], and non-small cell lung cancer [25]. Matsumoto et al. investigated the serum level of sIL-2R in 52 patients with RCC and 10 control subjects [26]. They also reported that measurement of sIL-2R in RCC patients provides useful information about the extent of disease and the survival time.

While sIL-2R is thought to be produced by tumor cells in patients with non-Hodgkin's lymphoma, the mechanisms responsible for the association between a high serum sIL-2R level and a worse prognosis of another malignancy have not been elucidated. IL-2 binds to its specific receptor (IL-2R) on target cells to induce various biologic changes. After being activated by IL-2, signaling via the IL-2R upregulates the expression of IL-2R*α*, partly through Stat5-dependant regulation of* Il-2r* *α* transcription [27]. Activities that require sustained IL-2R signaling are dependent on a continuing source of IL-2 to engage the IL-2R*α* and form additional IL-2/IL-2R signaling complexes. Therefore, when the *α*-chain of IL-2R is cleaved from the cell surface and released as the soluble receptor (sIL-2R), IL-2R signaling may be switched to the low signal pathway. Malek et al. reported that many of the key effects of IL-2 on Treg cells, but not Teff cells, require minimal IL-2R signaling, providing a therapeutic window to promote immune tolerance by using low dose IL-2 to act specifically on Treg cells [4, 6]. These findings may suggest that a relative decrease of IL-2R signaling due to an increase of sIL-2R induces Treg activation that promotes tumor immune tolerance and leads to a poor prognosis in patients with malignant tumors. Treg cells are a small subset of CD4^+^ T cells that can be identified by constitutive expression of CD25 (the *α*-chain of the IL-2R), and these cells control immune responses in multiple circumstances [28, 29]. Brivio et al. demonstrated a positive correlation between the serum concentration of sIL-2R and the number of Treg cells in patients with solid tumors [30]. The immunosuppressive state that occurs with enhanced Treg cell generation may be associated with an abnormally high serum sIL-2R level.

Activating the immune system via T cell regulation for the treatment of cancer has long been a target of studies on antitumor immunity. One of the most promising approaches to activating therapeutic antitumor immunity is the blockade of immune checkpoints. Because most immune checkpoints are initiated by ligand-receptor interactions, they can be readily blocked by antibodies or modulated by recombinant forms of ligands or receptors. CTLA-4 and PD-1 are major T cell regulatory molecules in the second signal of T cell activation that play an important role in tumor immune tolerance. There has been recent success with antibody-based strategies that enhance antitumor immunity by targeting T cell regulatory molecules. CTLA-4 antibodies were the first of this class of immunotherapeutic agents to achieve approval from the US Food and Drug Administration (FDA). Treatment with ipilimumab, a fully human monoclonal antibody that blocks CTLA-4 to promote antitumor immunity, induced a partial response in 5 out of 40 patients with metastatic RCC in a phase II trial [31]. In the present study, the serum level of sCTLA-4 was not related to the pathological grade, clinical stage, or prognosis of CCRCC. Although the IL-2/IL-2R signaling pathway is thought to be blocked by CTLA-4 [16], the serum level of sIL-2R was not correlated with that of sCTLA-4 in our patients with CCRCC.

Dong et al. reported that B7-H1 (PD-L1) is overexpressed by tumors both in vivo and in vitro, leading to immune tolerance, whereas blockade of B7-H1 or PD-1 enhances antitumor immunity [32]. An mAB targeting PD-1 frequently induces tumor regression in patients with advanced melanoma, RCC, lung cancer, and colon cancer while showing very low toxicity, and the response is correlated with tumor surface expression of B7-H1 [33]. Frigola et al. reported that elevated preoperative levels of circulating soluble B7-H1 (sB7-H1) were associated with an increased risk of death for CCRCC patients [34]. They mentioned that release of sB7-H1 molecules may be a tumor mechanism that impairs systemic antitumor immunity, suggesting that therapy which inactivates or removes sB7-H1 from the serum could be clinically beneficial. Crispen et al. reported that the immunohistochemical detection of B7-H3 expression by either tumor cells or tumor vessels was significantly associated with an increased risk of death from CCRCC [35]. Although the receptor for B7-H3 has not yet been identified, Prasad et al. demonstrated that mouse B7-H3 is a negative regulator of T cell activation and IL-2 production, and they concluded that B7-H3 regulates T cell proliferation via an IL-2-dependent mechanism [14]. The present study showed that the serum sB7-H3 level was significantly related to the clinical stage of CCRCC. In addition, this is the first report of a positive correlation between the serum concentrations of sIL-2R and sB7-H3 in patients with CCRCC to our knowledge. However, the present study enrolled a relatively small number of patients and the follow-up period was too short to draw definite conclusions. Prospective studies on a larger scale are needed in order to confirm the usefulness of measuring serum sIL-2R and sB7-H3 levels in patients with CCRCC as well as the relation between these two T cell regulatory molecules. Furthermore, if we accept the hypothesis that release of soluble T cell suppressor molecules is a tumor mechanism that interferes with systemic antitumor immunity, it is important to determine whether the serum levels of soluble T cell regulatory molecules such as sIL-2R and sB7-H3 can be used as biomarkers for predicting the effectiveness of immunotherapy targeting T cells in patients with CCRCC. It is hoped that further investigation of the promotion of tumor immune tolerance by T cell regulatory molecules will lead to the development of new immunotherapy options for CCRCC.

## 5. Conclusion

In the present study, the serum level of sIL-2R reflected tumor progression in patients with CCRCC, and the survival of patients with high sIL-2R levels was shorter than that of patients with low sIL-2R levels. The serum level of sB7-H3 was also significantly correlated with the clinical stage. Furthermore, serum levels of sIL-2R and sB7-H3 showed a positive correlation in CCRCC patients. These results suggest the possibility that the IL-2/IL-2R and B7-H3 pathways may be involved in the progression of CCRCC.

## Figures and Tables

**Table 1:** Characteristics of the patients with CCRCC.

CCRCC	Number = 70
Male/female	52/18
Mean age (range)	61 years old (40–84)
Pathological grade	
Grade 1	15
Grade 2	46
Grade 3	9
Clinical stage	
Stage 1	29
Stage 2	7
Stage 3	14
Stage 4	20

CCRCC: clear cell renal cell carcinoma.

**(a):** 

Serum levels	RCC patients	Control groups	*P* value
sIL-2R (pg/mL ± SD)	1080.2 ± 1251.3	587.4 ± 210.4	*P* = 0.043
sB7-H3 (ng/mL ± SD)	14.5 ± 10.1	19.3 ± 5.3	*P* < 0.0001
sCTLA-4 (ng/mL ± SD)	2.3 ± 0.7	3.0 ± 1.3	*P* < 0.001

CCRCC: clear cell renal cell carcinoma, sIL-2R: soluble interleukin-2 receptor, sB7-H3: soluble B7-H3, and sCTLA-4: soluble cytotoxic T lymphocyte associated antigen-4.

**(b):** 

	Grade 1	Grade 2	Grade 3	*P* value
sIL-2R (pg/mL ± SD)	767.0 ± 547.0	1141.8 ± 1464.4	1287.7 ± 815.3	*P* = 0.178
sB7-H3 (ng/mL ± SD)	13.2 ± 6.0	15.0 ± 11.5	13.9 ± 8.0	*P* = 0.946
sCTLA-4 (ng/mL ± SD)	2.2 ± 0.5	2.4 ± 0.8	2.1 ± 0.6	*P* = 0.533

CCRCC: clear cell renal cell carcinoma, sIL-2R: soluble interleukin-2 receptor, sB7-H3: soluble B7-H3, and sCTLA-4: soluble cytotoxic T lymphocyte associated antigen-4.

**(c):** 

	Stage 1	Stage 2	Stage 3	Stage 4	*P*-value
sIL-2R (pg/mL ± SD)	687.9 ± 497.4	725.1 ± 740.6	725.3 ± 460.9	2021.9 ± 1922.4	*P* < 0.001
sB7-H3 (ng/mL ± SD)	14.7 ± 12.4	10.2 ± 2.5	12.2 ± 6.9	17.4 ± 9.3	*P* = 0.044
sCTLA-4 (ng/mL ± SD)	2.2 ± 0.7	1.9 ± 0.3	2.5 ± 1.1	2.3 ± 0.6	*P* = 0.179

CCRCC: clear cell renal cell carcinoma, sIL-2R: soluble interleukin-2 receptor, sB7-H3: soluble B7-H3, and sCTLA-4: soluble cytotoxic T lymphocyte associated antigen-4.
